# Preparing for Practice: Evaluating a 3‐Week Longitudinal Ward Simulation

**DOI:** 10.1111/tct.70270

**Published:** 2025-12-09

**Authors:** Philip White, Jocelyn Amer, Adam Moxley

**Affiliations:** ^1^ Newcastle University Medical School Newcastle upon Tyne UK; ^2^ South Tyneside and Sunderland NHS Foundation Trust South Tyneside UK; ^3^ The Grove Medical Group Gosforth UK

## Abstract

**Background:**

Final‐year assistantships are a common feature of undergraduate curriculae, both in the United Kingdom and internationally. However, they often fail to adequately prepare students for practice, with task complexity and low confidence as common barriers to engagement. In the face of increasing burnout amongst newly qualified doctors, and recent studies showing lack of preparedness upon qualification, interventions to improve the efficacy and utilisation of assistantships are urgently required.

**Approach:**

A 3‐week longitudinal narrative simulation, framed within a virtual inpatient ward, was used to prepare final‐year medical students for assistantship. The simulation used persistent patient narratives that could be affected by student actions across a range of learning activities. Students had the opportunity to practice autonomously taking greater responsibility for meaningful decisions in handovers, patient admissions and discharges, on‐call and routine clinical tasks, emergencies and patient/family discussions.

**Evaluation:**

The module was evaluated with semi‐structured interviews and focus groups. Three main themes were identified: acting like a doctor, feelings of safety and feelings of legitimacy. Participants described the virtual ward as a stepping stone to participating on real wards during their assistantships by providing a safe but realistic environment to practice as an FY1, increasing self‐efficacy and self‐perceived professional identity.

**Implications:**

A longitudinal simulation using continuous patient narratives offered students realistic, consequence‐driven engagement with doctor‐level tasks, bridging the gap between classroom and clinical practice. Despite resource demands, this model may be a valuable tool to enhance assistantships, particularly in preparing students for roles typically inaccessible during training.

## 1 Background

Final‐year assistantships, designed to prepare medical students for practice, are a common feature of the UK undergraduate medical curriculum [1] and have been introduced internationally [2, 3]. These are heterogenous, with significant variations between medical schools, typically involving a late‐stage placement in a healthcare team, taking on the roles and responsibilities associated with Foundation Year 1 (FY1) doctors [4]. Assistantships have been criticised as often failing to effectively prepare students for Foundation placements [5, 6, 7], particularly feelings of empowerment, clinical reasoning, handover and prioritisation [7, 8]. During assistantships, students can find adopting the roles and responsibilities of the doctor challenging [7]. Struggling with low self‐efficacy may reduce self‐confidence, which further impacts engagement with assistantships [9].

*During assistantships, students can find adopting the roles and responsibilities of the doctor challenging* [7]. *Struggling with low self‐efficacy may reduce self‐confidence, which further impacts engagement with assistantships* [9].

In 2018–2019, the Newcastle medical school curriculum included a 3‐week module immediately prior to assistantships called ‘Preparation for Practice’ to prepare final‐year medical students for assistantship and work as FY1 doctors. The course was delivered at 10 hospital sites in the region. Sites shared learning outcomes and were largely classroom‐based. These outcomes covered many areas newly qualified doctors are often underprepared for, such as prescribing, handover and legal and ethical issues [5, 7, 9, 10]. Previous feedback for the module was mixed, with students failing to see the relevance of topics to their assistantships and future practice.

At one site, a longitudinal narrative simulation was introduced to contextualise the 3‐week module. Simulation is a common undergraduate teaching tool to help students progress up Miller's pyramid from ‘knows how’ to ‘show’ [11].

Simulation has a comprehensive evidence base supporting educational benefit [12, 13, 14], particularly in preparation for practice, since it provides a safe place to practice error reduction, hone technical and nontechnical skills and develop transferable skills for the real‐world setting. However, much traditional simulation is limited to a single session or day exercise, rather than an extended live‐action simulation of realistic physician work, which can limit the realism of a simulated scenario [15]. There are some examples of extended simulation in medical education. Laack et al. [16] conducted a 1‐week course on US medical students involving various patient‐care scenarios, showing increased self‐rated preparedness for practice via questionnaire. Rogers et al. [17] undertook a 1‐week longitudinal narrative simulation on Australian medical students with a similar format to ours, demonstrating increased knowledge scores on prescribing tests against a control 9 months later. Other examples of longitudinal simulation exist within other HCP professional education, demonstrating improved clinical competence [18, 19] and confidence in tasks or knowledge [18, 19, 20]. The format of these simulations differs substantially, with only a few including longitudinal narrative components that tie the learning experiences together.

*Traditional simulation is limited to a single session or day exercise, rather than an extended live‐action simulation of realistic physician work, which can limit the realism of a simulated scenario*.

Our simulation lasted 3 weeks and incorporated persistent patient narratives that could be affected in real time by student actions. The focus was on situations and activities directly relevant to students on assistantship, enabling them to meaningfully emulate a doctor's roles and responsibilities. This differs from traditional simulation, although it has some similarities to other extended longitudinal simulations, especially that by Rogers et al. [17]. Our study differs from other reported extended simulations through its qualitative data gathering methods, rather than self‐reported confidence surveys or changes in clinical knowledge. This project examined how far the intervention would bridge the gap between classroom, assistantship and clinical practice by allowing students to take responsibility for patients in a low‐risk environment and gain task and role confidence prior to assistantship.

*Our simulation lasted 3 weeks and incorporated persistent patient narratives that could be affected in real time by student actions*.

## 2 Approach

**FIGURE 1:**
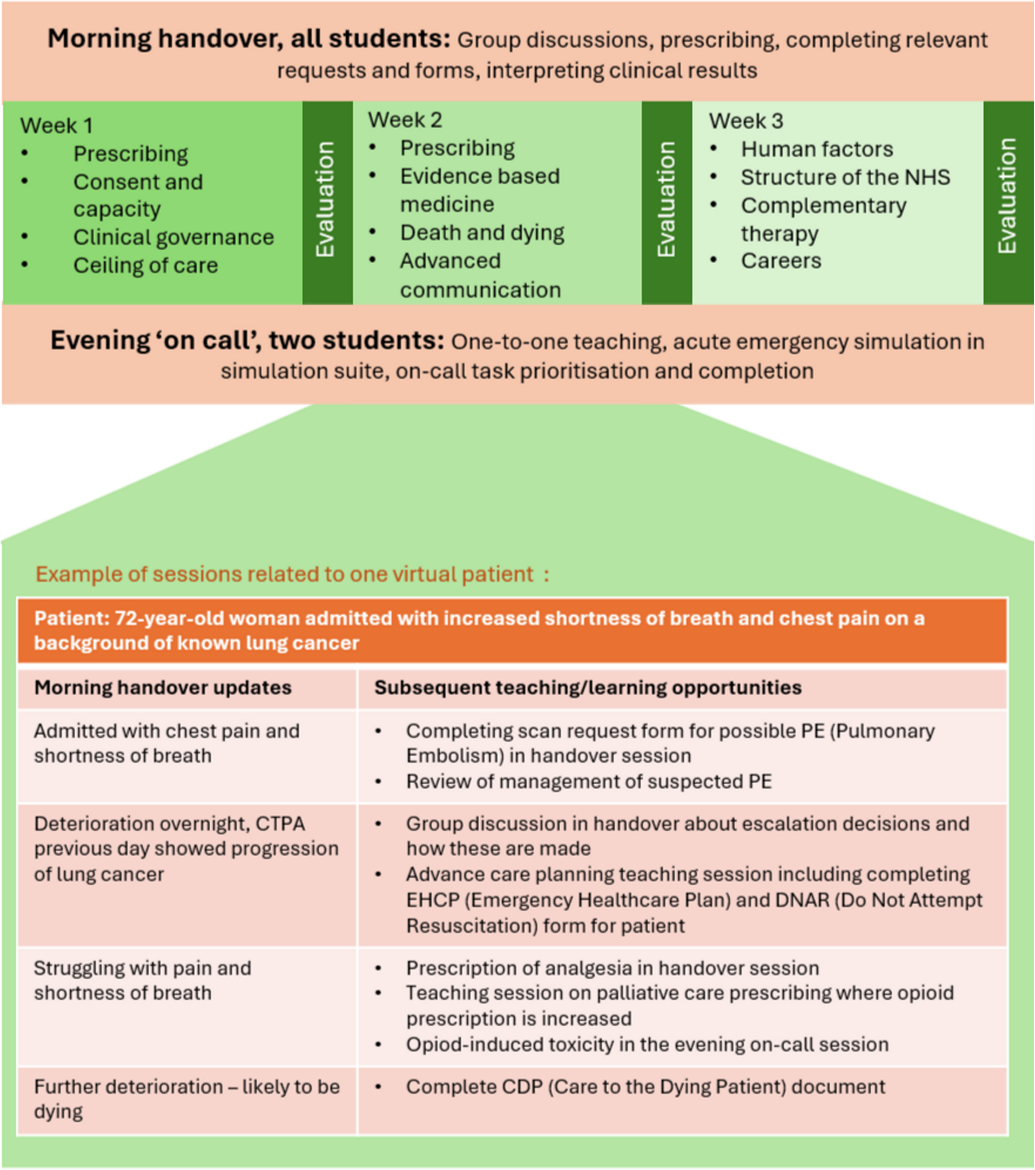
Structure of the module over 3 weeks and a sample patient.

A 3‐week longitudinal simulation was created with the same learning outcomes as other sites, reframed within a virtual ward populated with fictional patients and their families (Figure 1). It ran twice, in December 2018 and December 2019. Final‐year students acted as ward FY1s, whilst teaching fellows took senior roles. The simulation and activities focused on tasks that a student might encounter in practice or on assistantship.

Each evening, two students stayed for a simulated on‐call, individually practicing responding to an acutely deteriorating patient amidst routine on‐call tasks. These sessions included acute practical simulations with a high‐fidelity human‐like manikin, assisted by a teaching fellow who was role‐playing as a nurse. These acute scenarios included events that advanced different patient narratives. Support and feedback were limited until after completion (unless a student was struggling and requested assistance) to help build self‐efficacy. Mornings began with a handover, where on‐call students were responsible for updating the group with relevant information from the previous evening.

Daily learning objectives were based around patient narratives from handover, with fictional patients used as exemplars within sessions. Clinical reasoning ‘escape rooms’ involved clerking in new patients to the ward list, including phoning a medical registrar via hospital telephone systems. Communication role‐plays with actors were integrated into the narrative, covering tasks like breaking bad news, managing a complaint or discussing resuscitation that related directly to previous actions or events. Linked routine tasks such as discharge letters and requesting investigations deepened immersion and provided opportunities to develop task competence. The coherent narrative drew these different elements together, directly affected and altered by student actions. Further details from a how‐to handbook can be found in Appendix S1.

*The coherent narrative drew these different elements together, directly affected and altered by student actions*.

Collaboration with healthcare professionals is vital for adapting to professional practice. Pharmacists supported prescribing workshops, allowing students to ask practical questions as they might on the ward. Faith leaders and hospital chaplains led discussions on death and dying across spiritual traditions. Several of our ‘patients’ had different religious or cultural backgrounds, which enabled students to engage with faith leaders with specific, case‐focused questions rather than a more theoretical discussion. Though collaboration with nursing and paramedic colleagues and students would have been valuable, this was hampered by logistical barriers. Instead, teaching fellows and doctors role‐played other HCPs during simulations, from nurses in acute deteriorations to therapists in ward exercises. Students were able to work directly with the hospital switchboard by prior arrangement, directing phone calls to role‐playing teaching fellows. This included a simulated crash call alert during another routine task.

The simulation was evaluated via semi‐structured interviews and focus groups. Ethical approval was granted by Newcastle University. The 2018 cohort had interviews following their assistantships. Feedback from these interviews was used to develop and improve individual sessions, but no changes were made to the overall virtual ward structure. The 2019 cohort completed focus groups after their assistantships. Both cohorts were invited to follow‐up focus groups 1 year after programme delivery. All focus groups explored student experience of the module and its impact, if any, on their assistantships and early clinical practice. Qualitative data underwent thematic analysis [21] with codes developed independently by two researchers (PW and JA). Themes were generated through discussion and shared with participants for member checking.

## 3 Evaluation

Thirty‐four students undertook the curriculum. Twenty took part in the semi‐structured interviews and focus groups following their assistantship. Ten students took part in focus groups after 1 year.

**FIGURE 2:**
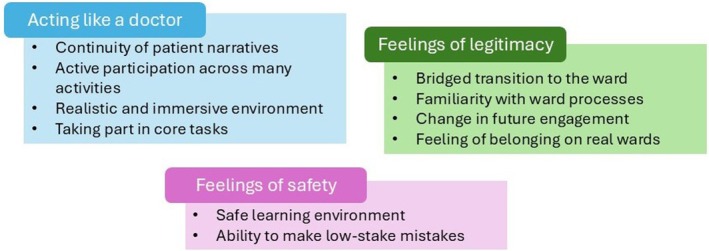
Themes and subthemes.

Three main themes emerged from the data—acting like a doctor, feelings of legitimacy and feelings of safety—with subthemes shown in Figure 2. Whilst the macro themes between 2018 and 2019 cohorts were very similar, any differences are indicated in the results.

### 3.1 Acting Like a Doctor

**FIGURE 3:**
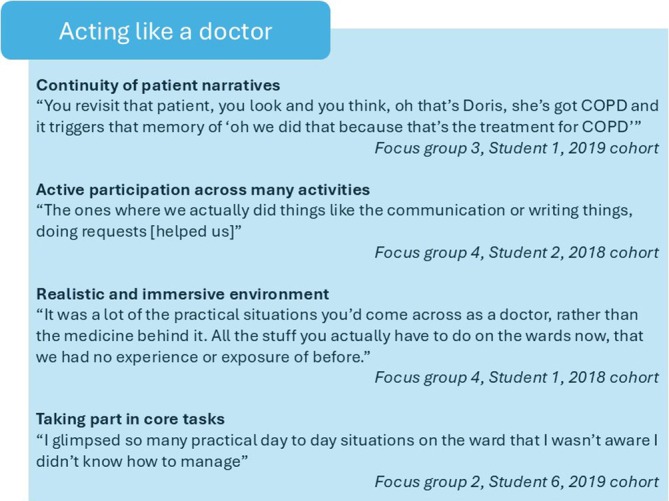
Subthemes from ‘Acting like a doctor’.

Four subthemes were identified under the theme of ‘Acting like a doctor’ (Figure 3). Participants described how the longitudinality of the patient narratives improved realism and immersion. In turn, this increased student engagement and enabled feeling legitimate responsibility for practical tasks. Some participants in the 2018 cohort credited this more to individual sessions than to patient narratives, particularly involving taking responsibility for, or enacting various clinical tasks such as phoning the registrar or prioritising on‐call lists. The 2019 cohort credited this more to the influence of the patient narratives.

*Participants described how the longitudinality of the patient narratives improved realism and immersion*.

### 3.2 Feelings of Safety (Figure 4)

**FIGURE 4:**
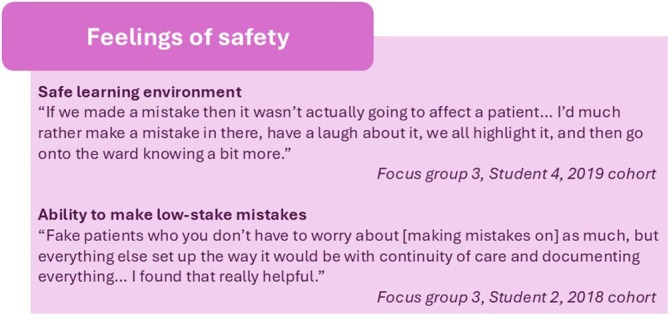
Subthemes from ‘Feelings of safety’.

Participants described the virtual ward as providing them with a setting in which they could learn from their errors. They felt that they could act more freely without the fear of mistakes and therefore felt more confident. Students particularly identified the benefit of being able to take responsibility for their decisions when they could see the consequences of these play out. Students formed and shared approaches to managing risk that they felt translated to clinical practice.

*Students particularly identified the benefit of being able to take responsibility for their decisions when they could see the consequences of these play out*.

### 3.3 Feelings of Legitimacy (Figure 5)

**FIGURE 5:**
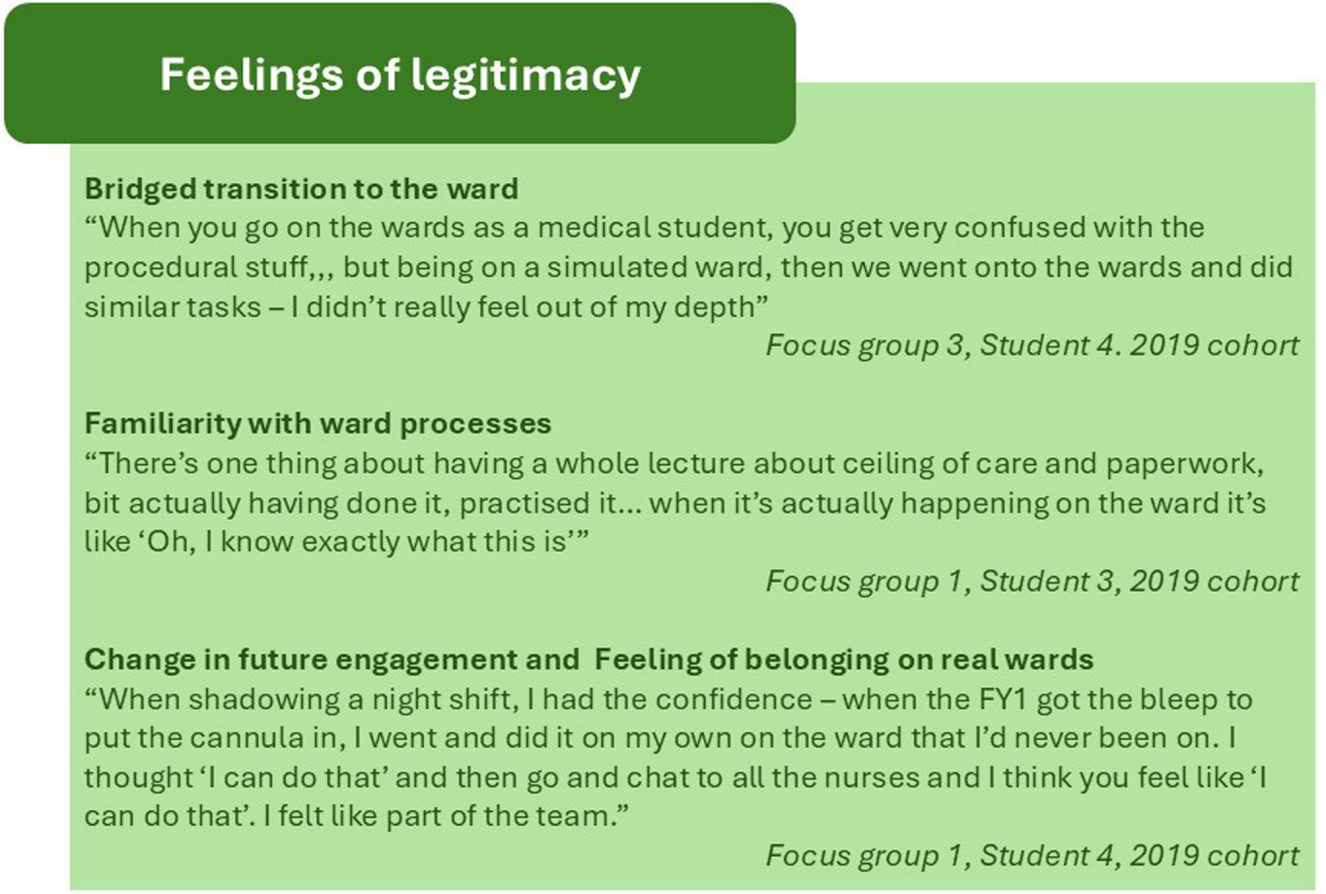
Subthemes from ‘Feelings of legitimacy’.

Participants described the virtual ward as a stepping stone to a more active role on the wards. Ward tasks and activities within sessions connected within a wider picture, which made carrying them out on a real ward easier and less daunting. Increased familiarity with basic tasks also enabled them to undertake more complex tasks once on real wards.

Some 2018 participants at the 1‐year focus groups reported little impact of the simulation on their assistantships—this was attributed by participants to their assistantships providing little opportunity for legitimate participation. However, all 2018 participants described the simulation as having had a positive impact on their self‐efficacy starting FY1.

*All 2018 participants described the simulation as having had a positive impact on their self‐efficacy starting FY1*.

## 4 Implications

The evaluation of this intervention shows that it successfully gave the participants the opportunity to act like doctors in an environment that felt safe for them to do so. This addressed known barriers to student engagement with assistantships [5, 6, 7] and increased self‐efficacy participating within medical teams during assistantship [7, 8].

The longitudinal nature of the simulation incorporating patient narratives and consequential actions meant that this simulation was able to address some of the limitations of traditional use of simulation where scenarios are disjointed from their wider context [15]. Where more traditional simulation elements were included, for example, the acute scenarios of deteriorating patients using high‐fidelity mannequins for the on‐call sessions, these scenarios were able to be grounded in a wider context with the students having already met the patient or would meet them again the following day.

*These scenarios were able to be grounded in a wider context with the students having already met the patient or would meet them again the following day*.

Some skills that may often be missing from typical simulation, such as the impact of ‘knowing’ the patients or managing a workload over time [22]. These skills were developed in our study through the inclusion of protracted and time realistic patient narratives and consequential actions for the students. Kinston [21] identifies how students may walk a fine line between too much and too little responsibility when taking on Entrustable Professional Activities. We felt the use of an extended simulation incorporating a virtual ward of patients where the students had the responsibility of ward F1 doctors, was integral in how students reported developing the mindset of a doctor.

Additionally, through the use of the virtual ward, the programme gave participants hands‐on experience with tasks they would not previously taken significant responsibility for, and often feel underprepared in [7, 8]. The safe environment increased the students' ability to participate in tasks confidently, resulting in increased engagement with similar tasks and in clinical teams during their assistantship [9]. However, this went beyond the benefits often associated with simulation. Students reported developing and sharing safe approaches through the longitudinal structure. By being able to ‘try these out’ in linked narratives, they were able to understand the simulated activities in their wider healthcare context. For example, conveying their own summaries of a patient journey across multiple clinical encounters to a senior rather than repeating a script from an instruction sheet.

*By being able to “try these out” in linked narratives, they were able to understand the simulated activities in their wider healthcare context. For example, conveying their own summaries of a patient journey across multiple clinical encounters to a senior*.

Our results add to the emerging body of literature on the impact of extended longitudinal simulation. Previous studies have demonstrated sustained improvements in knowledge, clinical competence and knowledge compared to controls [16, 17, 18, 19, 20]. This study uniquely gives qualitative insight into how the elements of the simulation affected student learning. For example, how the longitudinal narrative format develops skills around utilising patient familiarity to affect decision making, or how providing multiple ‘furnishing’ details of ward processes (e.g., calling switchboard, minor/practical patient queries from nurses) affects self‐perception and confidence engaging in practice by making the ‘unknown unknown’ known. It also aligns with other extended simulations by demonstrating a longitudinal impact 1 year later and by showing increased feelings of safety and reduction in possible errors [12, 14].

*The longitudinal narrative format develops skills around utilising patient familiarity to affect decision making*.

Limitations of this study include the small sample size at only one hospital site. Participants in the 1‐year follow‐up may have a selection bias; however, the only new data was about the impact of the simulation on starting the foundation programme. Our influence as researchers and teachers in this study may have affected data collection. One‐year focus groups were carried out with researchers who were unfamiliar to participants to minimise this. However, our grounding in the context of the data allows us to offer depth of insight into the results. The intervention is resource‐intensive to replicate, which is why we have included a handbook with many of our resources (Appendix S1).

Overall, this intervention shows that longitudinal simulations could be used as a tool to help prepare students for assistantships and FY1 by offering opportunities to access tasks that are important to the doctor role but not normally available to students and to give them the confidence to undertake these tasks within the simulation and subsequently in the clinical environment.

## Author Contributions

**Philip White:** conceptualisation, methodology, validation, formal analysis, investigation, data curation, writing writing – original draft, writing – review and editing, project administration. **Jocelyn Amer:** conceptualisation, methodology, validation, formal analysis, investigation, data curation, writing – original draft, writing – review and editing, visualisation, project administration. **Adam Moxley:** conceptualisation, methodology, investigation.

## Funding

The authors received no specific funding for this work.

## Ethics Statement

Ethical approval was granted by Newcastle University (14546/2018).

## Conflicts of Interest

The authors declare no conflicts of interest. All researchers were teaching fellows at South Tyneside Hospital Trust at the time of the study (P.W. 2018‐2019, A.M. 2018, J.A. 2019) and delivered and evaluated the curriculum.

## Supporting information


**Appendix S1:** Supporting information.

## Data Availability

The data that support the findings of this study are available from the corresponding author upon reasonable request.
