# Supplementary material for: Preparing for Practice: Evaluating a 3‐Week Longitudinal Ward Simulation
Source: Clin Teach. 2025 Dec 9;23(1):e70270. doi: 10.1111/tct.70270 (PMC12688705; doi:10.1111/tct.70270)
Supplement: Supplementary file 1 — Appendix S1: Supporting information. [file TCT-23-e70270-s001.docx]

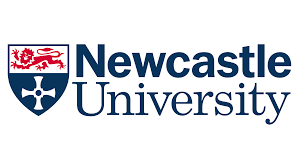


Handbook for creating a “Virtual Ward”

**Authors**

Dr Philip White

Dr Adam Moxley

Dr Jocelyn Amer

Dr Lucy Baxter

All authors were clinical teaching fellows at South Tyneside Hospital during the production of this document.

**Overview of Guide 3**

**Introduction 4**

Adapting for larger groups and other curricula. 5 The Patient Narrative 6 Handover 10 ‘On Call’ sessions 12 Clinical Reasoning 15

**Appendix A - Example Timetable 17 Appendix B - Example Handover Sheet 19 Appendix C - Detailed Handover Narrative 20 Appendix D - ‘On call’ resources 26 Appendix E - Simulation resources 33**

Overview of Guide

In 2018-19, Newcastle medical school had a curriculum called Patients, Doctors and Society 6 (PDS6) with a number of learning outcomes and classroom-based sessions focused around preparing students for assistantships and working as a doctor (F1). In previous years feedback nationally and locally, students often felt they didn’t feel ready for their assistantships or working as F1s. At our teaching site at South Tyneside hospital, we attempted to contextualise the outcomes and learning of PDS6 within a simulated ward to bridge the gap between student and assistantship. This included a daily handover, ward tasks and simulation as well as more traditional classroom learning.

Whilst this guide was designed for the Newcastle MBBS course PDS6 curriculum, we have written it so aspects of it can be adapted to any classroom-based curriculum.

How to approach this guide

The introduction outlines the overall aim of the PDS6 programme at South Tyneside hospital as well as covering the various types of the teaching sessions to contextualise the learning outcomes:

- Patient narratives
- Handover
- On-call
- Clinical reasoning

The appendices contain detail on how the timetable was structured and detailed plans / narratives for each day of teaching.

Finally, we have provided some resources for use such as a ward list template, simulation scenarios and on-call materials.

Introduction

We created a 3-week low-fidelity simulation to improve student confidence and self-efficacy in their assistantships and working as a doctor.

This was achieved by creating a ward of fictitious patients with continuous narratives that contextualised classroom-based teaching over the three-week period. The medical students were given the role of ward F1 doctors, with the teaching fellows taking the role of their SHOs or registrar.

Each day would comprise of a 1-hour handover in the morning during which patient events were updated and any requests / referrals were made on Newcastle Medical School paper stationary. Teaching throughout the day related to these patient events. Each afternoon between 4 and 5pm, two students were “on-call” and would prioritise and complete a list of ward tasks in addition to an acute simulation. Each simulation would either be a with a new ward patient or one of the current ward patients experiencing a deterioration.

In this guide, we have tried to break down each of the elements that went into creating this ward experience and have attached several templates and examples of our own resources in the appendices for use. Please see each section for a more detailed break-down of these.

Adapting for larger groups and other curricula.

In 2018-19, PDS6 was the last classroom-based rotation at Newcastle before students completed their degrees. It was located just before Christmas, preceded by clinical attachments in Mental health, Paediatrics, Obstetrics and Gynaecology and General Practice. It was followed by clinical apprenticeships in medicine and surgery. There was a wide range of subject matters covered, from death & dying to clinical reasoning and complementary medicine.

We feel that creating a ward simulation is an effective way of framing classroom teaching for students in their latter years of study, particularly given how much students felt this prepared them for life on the wards afterwards. It was highly engaging and immersive for students, not to mention being enormously fun and rewarding to deliver! Our evaluation showed that it helped enhance the students self-perceived professional identity, self-efficacy, sense of belonging in real ward teams. Further, students reported that they felt able to take on legitimate ward roles and actions in a way where mistakes were low-stakes and could be learned from without fear of real-world impact on patients. We would recommend adopting aspects of this approach to any fully or partially classroom-based curriculum for more advanced students.

At STGH, only 16-18 students per cohort attended for PDS6, although group sizes in other hospitals varied from small groups to over 100 students. We recognise that the outlined strategy for contextualising teaching may be easier to implement on a smaller scale.

However, we feel that certain elements of this approach may still be readily applicable for larger group sizes. Some of the elements described are faculty or resource intensive. For example, simulation sessions ideally require the use of a simulation lab and at least two teaching staff to run. Sites with higher numbers of students may struggle to provide an equitable experience for all students due to time and resource constraints in simulation. However, they may still be able to adapt other elements such as the patient narrative or ‘on call sessions’ effectively (please see below).

With regards to each theme outlined below, we have described what we did and suggested how this may be adapted for smaller groups or larger groups.

The Patient Narrative

*What we did*

Our simulated ward had a total of 17 patients admitted or discharged over the 3-week period. Each patient was used to illustrate or contextualise one (or several) learning points. Patients were ‘admitted’ either via an acute simulation during the on-call sessions, through clinical reasoning cases or through the morning handover.

‘Patients’ would follow a progression in real-time, reflective of reality, often requiring tests or interventions to be requested or interpreted by students. Some patients would be discharged, allowing discussion around discharge plans and for students to practice writing discharge letters. Other patients, often with more complex needs stayed the entire three weeks leading to discussion around what may keep patients in this long and complications of prolonged hospital stays. Several patients passed away under different circumstances, tying into the death and dying portion of the PDS6 syllabus.

There is no arbitrary order in which PDS6 sessions must run, so we aligned sessions with patient stories, sometimes creating event chains that meant students became more invested in their ‘patients’, meaning that their actions may even have consequences.

-------------------

***Example 1***

*On day one, students are given a list of patients on their ward with space to write notes for themselves about each patients. During handover on day two, we handed over to students that a new patient, Ella Jones, has been admitted with a stroke. Students are instructed to take a handover and encouraged to ask clarifying questions as they feel are needed. Students are told she requires an NG tube for feeding, having lost her ability to swallow, and need to request an Xray.*

*Activity 1: students request a chest Xray using exam stationary. Activity 2: students must interpret the chest Xray in pairs, documenting their findings in the notes.*

*We complicated this task for students, by having a patient in the next bed (already on their patient list, but not drawn attention to) called Emma Jones with similar demographics. This patient had dementia and had also had an NG tube placement with confirmatory chest Xray already requested. Students were initially shown Emma’s Xray (correct placement) with her patient details written in the top corner and only allowed to see Ella’s Xray (incorrect placement) if they spotted they were looking at the wrong patient’s Xray.*

*That afternoon, the ‘on call’ student is asked to come and see Ella Jones for an aspiration pneumonia in the acute simulation. Unfortunately, nurses misheard the instructions and removed Emma’s NG instead, feeding Ella. The next morning, on day 3 the student on call the previous afternoon must handover these events to the rest of the group.*

*During handover on day 4, the students must read out an angry letter from Ella’s son, Giles, a medico-legal prosecution lawyer. Teaching that day is on medical error, human factors, clinical governance and never events with each session loosely linked to Ella’s situation. On day 5, students are taught about quality improvement projects and are tasked to design a systems-based solution to prevent this medical error from happening again. On day 7, the students then get to meet Giles face to face during an advanced communication session where an actor plays the son. Students commented on how much more real and intense this experience felt in the context of all that had happened beforehand.*

-------------------

A further example of how we used the narrative to improve the authenticity of the communication stations is discussed below:

***Example 2***

*During the afternoon on-call session on day 10 (Theme: Death & Dying), students are told that they are on a night shift and the time is 6am. They are called urgently to the emergency room to deal with an acute trauma case of a young man, Dave Woodford, who has been in a car accident and has a decreased Glasgow Coma scale after a head injury. They stabilise him during the simulation.*

*During handover the next morning (day 11), they must request a CT pan trauma scan. We asked switchboard to send ourselves a simulated crash call to our bleeps halfway through the session and both ran out of the room as if responding to an arrest. A few minutes later, we came back in and told students that Dave Woodford had sadly passed away on the way to his CT and we were unable to resuscitate him.*

*Students were then told that his family, some of whom have come in from home (mother, grandma, wife), and his aunt who was driving Dave back from a concert at the time are all outside and that the students need to break the bad news of Dave’s death to them. His family were all played by actors. Each member of the family had a different reaction to the news (silence, anger, tears, denial) that students had to grapple with.*

*Whilst this may have seemed a rather intense way to throw students into the session, we prepared the students with plenty of signposting for pastoral support and debrief if they found this impacted on them significantly. We felt that it was important that students be placed in a situation they had to tell someone that they (or a relative) will die/has died in an artificial situation rather than a real one for the first time.*

-------------------

Suggestions:

*Tips for practice*

- Use themes of teaching sessions to link together a story for several patients that students can follow through the 3-week period
- Involve those who deliver sessions to try and get as much of the content linking back to the narrative of the ward (case-studies etc.)
- Have a separate Excel sheet file with a clear daily narrative side by side with sessions to be covered so information retains continuity and coherence. Make this available to all staff involved with programme delivery.
- Have demographic details of patients and any details you reveal to the students (i.e. conditions, PMH, DH) stored on a separate Excel tab and make this available to all teaching staff.
- It may sometimes be easier to create a new patient than to have multiple themes running through one patient’s journey. Separate patients may help concepts stick in students minds – i.e. one patient to represent medical error, one for palliative care etc.
- We suggest having no fewer than 10 patients but no more than 20.
- Linking similarly themed concepts together in one narrative may make it more engaging or believable e.g. DNR, breaking bad news, death and dying, cancer journey & alternative therapies can all link coherently across one narrative.

*For larger groups*

- Decide on what level of immersion you would like to create before you start planning.
- Ensure you effectively communicate this concept to all staff taking part in teaching delivery beforehand and be clear about how much you want their session to link in.
- Only about half of our sessions linked directly to the narrative, and students still found this to be a very immersive experience.
- You may just want to create a ‘Handover slot’ at the beginning of the day in which all events and related exercises take place. You can then signpost that the sessions covered that day will relate thematically to events discussed in Handover, even if they don’t refer directly to them.
- Consider splitting students into small groups of between 4-8 for the course of the curriculum and each managing the ‘ward’ within these groups.

Handover

*What we did*

Students would arrive at 9am. All students would be given a ward list (see Handover sheet, Appendix B), receiving a fresh copy each week (it would become too messy/complex otherwise). Two students (whoever was ‘on call’ the evening before) would arrive at 08:50 and be given the task of handing over the events of the previous night. They would prepare the ward list updating jobs and patient events from the ‘on call’ session the night before. At 09:00 they would then divide the list between them and handover the ward to other students who would have to write down what they had heard. Feedback/discussion would be facilitated by a teaching fellow with tips given for improvement of Handover technique (i.e. useful/unnecessary information, encouraging receiving students to ask more questions).

9:15 - 10:00 usually involved 1-2 focused activities, usually along a prescribing or data requesting/interpretation theme. This may involve prescribing blood, drugs, ordering investigations on exam stationary, filling in/discussing scoring systems ie. GRACE, Blatchford/Rockall, Wells, filling in DNR or death certificates or filling in a discharge summary. All activities would be linked to the patient narrative (above) and would usually have a consequence later (i.e. tests requested then had results coming back during the on-call session and would need interpreting and actioning).

***Example 3***

*We filmed a mini ward round from a first-person point of view with a teaching fellow acting as specialist registrar and admin staff in the roles of 3 different patients from our narrative. We provided students with continuation sheets then showed them the film, asking them to each document as if it were a ward round. ‘Gold standard’ documentation written by teaching fellows was distributed to students afterwards, with a time to discuss what the students had written and compare.*

Suggestions

*Tips for practice*

- Don’t try and overfill this slot. Having only one or two activities planned means plenty to time to discuss any issues that crop up. Feedback is key to learning! Spare space can be facilitated with discussions around events from the previous day.
- Keep to time with the handover slots to avoid overrunning.
- We found that asking the students handing over to come at 8:50 to prepare their handover for a 9am start was more effective as it encouraged all other students to arrive at 9am rather than miss the handover.
- To help align our ward ‘story’ with appropriate teaching materials we used the university list of exam stationary combined with important scoring systems often used in practice, then fitted this in either with patients we already had on the ward or created new patients to fit the story.

*For larger groups*

- This activity could easily be scaled up for larger groups of students, by either replicating the above model and breaking up into groups of around 8, or if there aren’t enough faculty, students could be given the opportunity to go through the materials by themselves or in pairs with a model answer being demonstrated in a lecture style.
- Students seemed to most value personalised feedback with investigation request forms as they were often not confident with filling this in. Making each area of the exam stationary as explicit as possible during feedback was essential (including patient demographics/tick boxes etc.). This would likely need extra emphasis in large groups when demonstrating if you do not have the faculty to give individual feedback.

‘On Call’ sessions

*What we did*

We finished our formal mandatory teaching time at 4pm every day. Each day, two students would be assigned the role of being ‘on call’. This would take place in 3 phases:

1. **Handover & prioritisation**Students would start in the education centre where a teaching fellow would give them a pair of bleeps and a small list of jobs (~4) to complete between them. This would be given in the format of an in-person F1 from the dayshift (a teaching fellow) handing over outstanding jobs to an F1 out of hours (the students). Students would be expected to question the faculty member further for more detail about the jobs, then would be required to start prioritising in what order they would carry out the jobs. During this time, they would each be bleeped by another member of faculty posing as a nurse. One of these jobs would be another on-call task that they would have to re-evaluate their priority list for, whilst another would be a call to see an acutely unwell patient (again, to be evaluated in their priority list).
2. **Acute simulation and debrief**

One student (A) would attend the acutely unwell patient by completing a 10-minute simulation station (either a patient on the ward with worsening of their presenting complaint or a new patient in the emergency room). Both teaching fellows would be required for this, with one operating the sim man doll and the other acting as a nurse to assist. Around 20 minutes would be devoted to a 1-1 debrief with the student around the simulation, focusing on whatever the student/faculty agreed was most challenging about the scenario. One faculty member would be required for this, whilst the other would go and debrief with student B. During this time, student (B) would go to a separate room where each of the jobs from handover would be laid out in paper form. They would be instructed to complete half of them (2-3) without any help close to hand.

1. **Job completion and debrief**

Once the student had completed simulation, both students would swap. Student B would now undertake the same simulation session (above). Student A would have to complete the other half (2-3) of these jobs using only the information made available to them from handover. They would typically have about 15 minutes for this. The faculty member no longer helping with the simulation session would then come through and debrief both the thought process of prioritisation/gaining information from handover and go through the jobs with student A. Any issues would be discussed and the session would finish shortly after 5, depending on how long debrief took.

***Example 4***

*During the morning Handover session on day 6, Anya Polanski, our lung cancer patient was complaining of increased pain. We spent the session discussing increased dosing of opioids and working out breakthroughs. That evening, the students are given a list of jobs: insulin prescribing, reviewing an agitated patient, prescribing fluids, prescribing an anti-emetic (see appendix D for further details of each). Whilst deciding in which order to see the patients, student A received a bleep from a faculty posing as an Emergency Room Doctor with a handover for a patient called Abena Mensah coming up from the Emergency Room, asking for them to prescribe appropriate antibiotics for a pneumonia. Student B then received a bleep from a nurse on our ward, concerned that Anya Polanski is very drowsy and unresponsive.*

*The simulation focused on ABCDE assessment of a patient with opioid overdose, and student B would be provided with some patient details including the drug charts that they (themselves) had filled in increasing their opioid prescriptions that morning. Student A (who took the handover) went to the second room for on-call tasks to prescribe the antibiotics for the other handed over patient, and complete the two jobs they felt most urgent on the list handed over by the faculty member at 4pm. At 4:30, both students swapped.*

*Unknown to student A, Abena Mensah is penicillin allergic (not documented or handed over) and so their simulation is dealing with the anaphylactic reaction from penicillin antibiotics. A learning point to be discussed would be always to check with the patient if they have any allergies, even if ‘No known drug allergies’ is documented. Student B would continue completing the remaining two jobs in a different room.*

*Both students would be expected to handover* ​*relevant*​ *information to their colleagues the next morning.*

Suggestions:

*Tips for practice*

- Ensure you have rooms booked well in advance. This exercise requires three rooms: one to take the handover, one to complete the jobs and one for Simulation. However, the handover room and the job-completion room could be combined.
- These sessions can comfortably be handled by only two faculty members, so long as they are both clear on what will happen. One member can prepare the simulation and jobs completion area at ~3:30pm then bleep the students at 4pm; whilst the other hands over the information to the students at 4pm.
- It is worth having mobile contact between both faculty during the session as co-ordination of timings may be difficult without this.
- The same (or similar) list of on-call tasks can be used for each student pair. Creating one master copy of the materials and photocopying these saves a lot of work! (though leaving the date box blank and updating each with the date of the exercise may increase authenticity of the experience)
- It may be worth considering not scheduling on-call immediately after more emotive seminars such as death and dying.

*For larger groups*

- If faculty/rooms are limited, consider scheduling on-call sessions at lunchtime as well, or two sessions at 3pm and 4pm.
- The jobs list/prioritisation exercise (and completion) was a well rated/useful exercise for students without the SIM session. This could be scaled up to have groups of 5-10 students ‘on call’ prioritising and completing jobs lists individually before coming back together for a small group debrief of the session.

Clinical Reasoning

*What we did*

Clinical Reasoning sessions have been used throughout the region in different formats by teaching fellows for a few years. Whilst we did not specifically create them for PDS6, they were particularly highly rated sessions and provided additional tie-in to our narrative, adding to the immersive experience. We found the students really valued the integration of clinical skills and knowledge with the non-technical skills outlined in PDS6. Students commented that this grounded the importance of these in practice.

These sessions required a bit of advanced preparation and worked a little like a medicine-themed escape room - but whilst developing their clinical reasoning! Students would be placed in groups of 4 and given a completed admissions clerk in document. They would then have to fill in separate summary pages, suggesting their impression, plan and creating a jobs list. This jobs list would then be actioned in real-time - all requests would be made on exam stationary and handed to faculty, who would then vet the request (bounce it if inappropriately filled in) and respond by providing the students with the test results requested. Additionally, if they needed to call a senior, ask a nurse to do additional tests or get a collateral history, a number would be provided for them to call (a member of faculty) who would then respond appropriately over the phone.

Suggestions:

*Tips for practice*

- Ensure you have all your resources printed and clearly prepared/organised before delivering the session. Consider whether the students are likely to ask for CT scans, Chest xrays, ECGs etc. These sessions tend to get a bit hectic, so a clear system to process requests is advised!
- Don’t get too flustered if you get a wave of requests at the same time. Ideally, these sessions run best with two faculty, but you can prepare students for delays by advising that this may be more reflective of real-life, and they could still continue to develop other areas of their management plan.
- Cheating (getting results off other teams) did occur a few times - this could be reduced by placing groups in separate rooms or making the exercise into a competitive race so that groups are less likely to share information.
- Consider ways to make these sessions more electronically based rather than paper based (for environmental reasons at least!). If you come up with any bright ideas, please let us know!

*For larger groups*

- These sessions scale as far as you have faculty to deal with requests and room to host small groups. The more students however, the more photocopying may be involved... (unless an electronic alternative is created)

Appendix A - Example Timetable

A broad overview of the first two weeks of PDS6. For more detailed handover narrative, please see Appendix C (each day is numbered).

Week 1

|  | **Theme** | **Handover**  **09:00 – 10:00** | **Teaching**  **10:00 – 16:00** | **On-call simulation**  **16:00 – 17:00** |
| --- | --- | --- | --- | --- |
| **Day 1** | Introduction to PDS6  Microbiology | - Introduction to PDS6 and “ward” - Handover of all current patients - Prescribe antibiotics for patient with community acquired pneumonia | - Introduction to simulation and SimMan - Allocation of complementary therapy poster topics - Microbiology guidelines - Prescribing workshop - Antibiotics | Self-directed study |
| **Day 2** | Capacity, Consent and the Confused patient | - Confusion screen bloods - NG tube chest x-ray placement - Deprivation of Liberty form | - The confused patient - Consent and capacity | Upper GI bleed |
| **Day 3** | Blood transfusion | - Request OGD - Request blood products | - Blatchford score - Blood transfusion - Prescribing workshop – blood, anticoagulants | Pulmonary embolism  Aspiration pneumonia |
| **Day 4** | Communicating risk  Never events | - Request CTPA - Calculate​ ​Wells score - Antibiotic prescription - Read out angry letter | - Medical error - Human factors - Clinical governance and never events | Diabetic Ketoacidosis |
| **Day 5** | DNACPR and Advance care planning | - Complete DNACPR - Complete EHCP | - Ceilings of care / DNACPR - Prescribing workshop - insulin and fluids | Self-directed study |

Week 2

|  | **Theme** | **Handover**  **09:00 – 10:00** | **Teaching**  **10:00 – 16:00** | **On-call simulation**  **16:00 – 17:00** |
| --- | --- | --- | --- | --- |
| **Day 6** | Evidence-based medicine  Quality Improvement | - Ward round video - documentation | - Prescribing workshop - palliative care - Evidence based medicine - Quality improvement | Lung cancer opioid overdose Anaphylaxis from penicillin allergy |
| **Day 7** | Palliation Discharge planning | - Write discharge summaries - Group situational judgement discussion with alcoholic patient wanting to self-discharge | - Discharge planning - Advanced communication (including complaint and alcoholic patient self-discharges) | Alcoholic patient readmitted with seizure/low GCS |
| **Day 8** | Biopsychosocial model | - Managing alcohol detox - Electrolyte replacement - End of Life documentation | - Biopsychosocial model - Clinical Reasoning - Prescribing workshop - electrolytes | New patient with fast atrial fibrillation |
| **Day 9** | Complaints Shared Decision Making | - Anticoagulants - CHADSVasc score - HASBLED score | - Mock root-cause analysis - Shared decision making | Road traffic accident/head injury brought in |
| **Day 10** | Death & Dying | - Referrals - Doppler/CT angio | - Death cafe - Advanced communication 2 –breaking bad news - Managing death as a junior Doctor | Self-directed study |

Appendix B - Example Handover Sheet


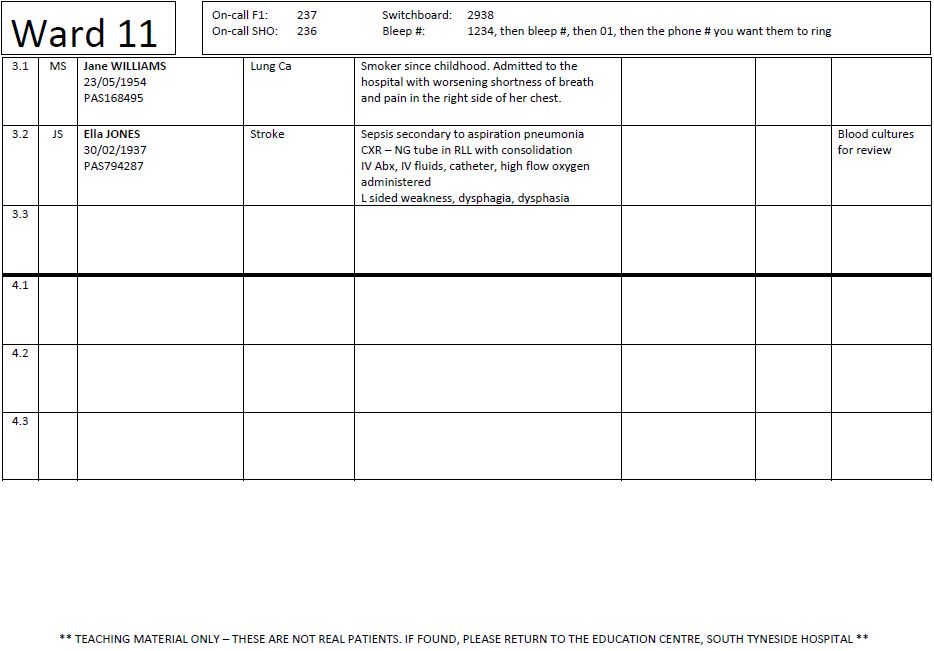

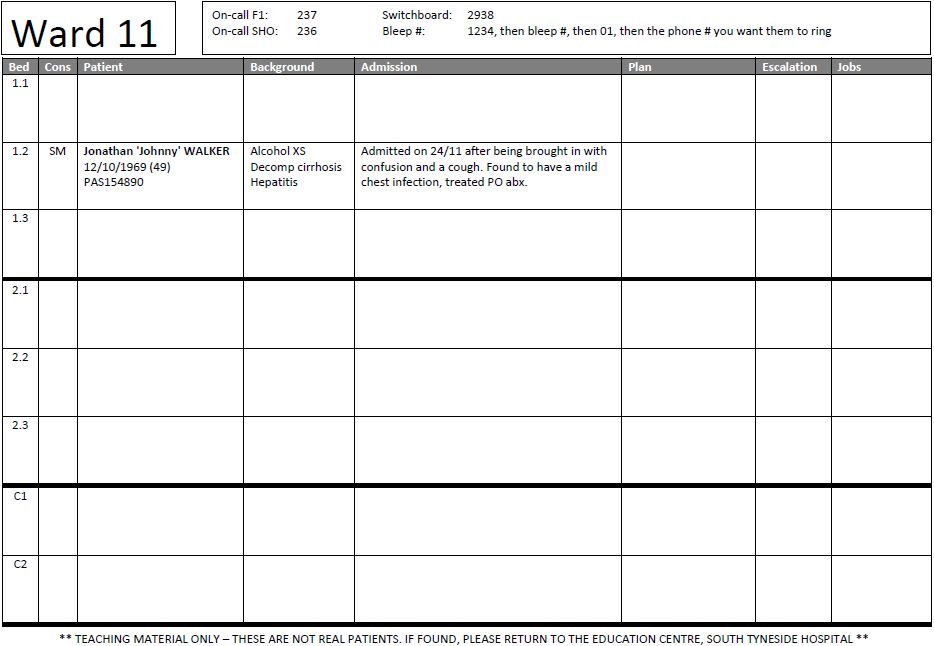
Please see other attachments for master-copy to use.

Appendix C - Detailed Handover Narrative

Day 1

| **Ward patients**   - Jonny Walker - alcohol excess - Anya Polanski - lung cancer - Ella Jones - stroke with aspiration pneumonia | **Discharged**  None |
| --- | --- |
| **Handover**  Introduce students to the new ward and the current patients | |
| **Tasks**   - Orientate students to handover sheet | **Materials**   - Ward handover sheets for each student |

Day 2

| **New patients**   - William McAllister - Bed 1:1 - Chukwuedu Achebe - Bed 1:4 - Emma Jones - Bed 3:3 | **Discharged**  None |
| --- | --- |
| **Handover**   1. Johnny Walker seems more confused 2. NG tube in place for Ella Jones so we need to sign that off 3. Emma Jones keeps trying to leave the ward – do we need to think about a Deprivation of Liberty (DoLs)? | |
| **Tasks**   - Confusion screen for Johnny Walker - Request chest x-ray (in retrospect) for William McAllister - Review x-ray for Ella Jones   - Give students Emma Jones’ chest x ray - she has a correctly placed NG tube   - If noticed, provide Ella Jones’ x ray which has misplaced NG tube - What do we do about a DoLs? | **Materials**   - Blood request forms - Radiology request forms - Chest x-rays   - Ella Jones   - Emma jones - Copy of notes for Emma Jones   (continuation sheet with demographics at the top) |

Day 3

| **New patients**  None | **Discharged**  None |
| --- | --- |
| **Handover**   - - 1. Johnny Walker has had an upper GI bleed overnight | |
| **Tasks**   - Discuss plan for Johnny Walker - Calculate Blatchford - Request OGD - Request transfusion bloods | **Materials**   - Blood request forms - OGD request forms - Blatchford score proformas |

Day 4

| **New patients**  None | **Discharged**  None |
| --- | --- |
| **Handover**   1. Anya Polanski has had a pulmonary embolism - she wasn’t on prophylactic enoxaparin. Radiologist wanted to wait until morning for CTPA, so we need to book it. 2. Ella Jones became septic with a chest infection last night. We think is an aspiration pneumonia. Nursing staff informed last night and we got a letter this morning from her son (Giles Jones, who also happens to be a lawyer). | |
| **Tasks**   - Read complaint letter and work out how Ella had an Aspiration Pneumonia - Request CTPA - Calculate​ ​Wells score - Antibiotic prescription | **Materials**   - Complaint letter - Drug charts - Radiology request forms |

Day 5

| **New patients**   - Mohammed Iqbal - Bed 1:3 | **Discharged**  None |
| --- | --- |
| **Handover**   1. New DKA patient (Mohammed Iqbal) 2. Lung cancer patient still short of breath and increasing oxygen requirement    1. Need to think about escalation | |
| **Tasks**   - Complete DNACPR and EHCP (after short teaching session) | **Materials**   - DNACPR forms - EHCP forms |

Day 6

| **New patients**   - None | **Discharged**   - None |
| --- | --- |
| **Handover**   1. Lung cancer patient (Anya Polanski) has read up on new immunotherapy treatment in the United States and has asked if we can trial them on it. Her daughter has asked if we can start more antibiotics as surely this will help her shortness of breath. 2. We think Johnny Walker has had someone bringing alcohol in for him over the weekend as a maintenance engineer found three bottles of cider in the ceiling tiles above his bed. | |
| **Tasks**   - Observe filmed ward round, practice documentation - Anya Polanski is in pain - increase opioid dose on drug charts - What do we think about patients who drink/smoke on the ward? What is a good response? (situational judgement discussion) | **Materials**   - Ward round film - Continuation sheets for Documentation - Drug charts |

Day 7

| **New patients**   - Abena Mensah (penicillin allergic patient given penicillin the night before) | **Discharged**  None |
| --- | --- |
| **Handover**   1. New patient (Abena Mensah) admitted yesterday for UTI given stat gentamicin and co-amoxiclav but was penicillin allergic and had successfully treated anaphylaxis in the Emergency Room. She is not aware of why this happened. 2. Charlie Brown settled over the weekend- seen by Endocrinology and ready for discharge. 3. William McAllister settled, ready for home today. 4. Anya Polanski has worsening cancer on CTPA - needs discussion regarding ceilings of care and breaking bad news. May be suitable for hospice. 5. Johnny Walker getting upset and has asked to talk to a doctor about wanting to go home.   *(This handover information largely sets up the context for the advanced communication stations happening later that day)* | |
| **Tasks**   - Discharge letters for William McAllister or Mohammed Iqbal - Hospice referral for Anya Polanski | **Materials**   - Continuation paper - History/information for students on patients being referred/discharged |

Day 8

| **New patients**   - Jonny Walker (readmitted) | **Discharged**  William McAllister, Charlie Brown, Jonny Walker (then later readmitted last night) |
| --- | --- |
| **Handover**   1. Johnny Walker readmitted overnight with seizure with deranged electrolytes: magnesium 0.47, phosphate 0.41, potassium 2.6, sodium 128, calcium 2.2. 2. Anya Polanski deteriorated overnight, decision made for end of life care. | |
| **Tasks**   - Initiate End of Life documentation - Pabrinex and CIWA prescription - Electrolyte replacement | **Materials**   - Care of the Dying pathways - Drug chart |

Day 9

| **New patients**   - Henry Wellington (Post upper GI bleed - high potassium) - Ma Cheng (Decompensated alcoholic liver disease, diarrhoea - low magnesium) - Cheun Ng (Infective exacerbation of COPD - low potassium post salbutamol) - Mavis Turner (Generally unwell - low sodium ?cause)   *(All patients admitted through yesterday’s electrolytes prescribing workshop)* | **Discharged**  None |
| --- | --- |
| **Handover**   1. We need to start anticoagulation on Christine Wooler. 2. Management following up on NG tube feeding error- would like to have a meeting at lunchtime ​*(set up for mock root-cause analysis)* | |
| **Tasks**   - CHADSVASC - HASBLED - Prescribe warfarin - INR check and prescribe next dose | **Materials**   - Drug chart - Phones/sheets with bleeding scores available |

Day 10

| **New patients**   - None | **Discharged**  Ma Cheng, Henry Wellington |
| --- | --- |
| **Handover**   1. Anya Polanski’ family asking how long it will be until she dies? She has been on the end-of-life pathway for a few days. They have been in nearly 24/7 since last Tuesday. Hospice have agreed to take her today. 2. Over weekend, Christine Wooler has cold, painful leg, very weak foot pulses. | |
| **Tasks**   - CT angiography legs request - Vascular surgeons referral - Chaplain’s questions (chaplains to come in on day 11 to discuss different cultural/religious perspectives on death and dying - students to submit questions for Q&A) | **Materials**   - Radiology request forms - Continuation paper - Post-it notes |

Day 11

| **New patients**   - Dave Woodford | **Discharged**  Anya Polanski - to a hospice |
| --- | --- |
| **Handover**   1. Anya Polanski transfer got delayed until later this morning. 2. Vascular came to RV Christine Wooler- scan showed partially occluded arteries in legs. Will see in OP. 3. Pre-alert early this morning, Dave Woodford. Just been stabilised, needs CT pan-scan trauma urgent. | |
| **Tasks**   - CT pan scan - GCS calculation - Practice a log roll - *Fast bleep to an arrest – see example 2* | **Materials**   - Radiology request forms - Willing student for log roll - Bleeps |

Appendix D - ‘On call’ resources

| **Insulin Prescribing** | |
| --- | --- |
| Task description: | Student asked by nurses to prescribe insulin dose for patient for that evening and next morning/day |
| Materials required: | - Insulin chart - Clerk-in notes with insulin dose (optional) |
| Outcome: | - Student should recognise low blood sugar (BM) from evening before and reduce morning (not evening) dose by 10%. - Can be handed over that insulins need prescribing in the day and reduced dose. |
| Comments: | This requires a pre-written insulin chart with doses given from previous day, and BMs showing an evening hypo but normal morning BMs. This can be filled in once (bar filling in dates), then photocopied for as many students/days as is needed and dates filled in afterwards. |

| **IVT Prescribing** | |
| --- | --- |
| Task description: | Student asked by nurses to prescribe IV fluids (IVT) for a patient |
| Materials required: | - One-sided history sheet containing full assessment and exam details of patient (recent U&Es, clinical fluid status, signs of overload, history of why needs IVT) - IVT prescribing sheet - Fluid balance sheet |
| Outcome: | - Students should weigh up information in history and prescribe fluid appropriately. Specify which fluid and appropriate rate. - Can be handed over the next morning re: checking fluid balance |
| Comments: | This requires a written history with all relevant information (students do not have a patient to examine) and fluid balance sheet filled in with urine output/fluid input from the last 24hrs. This can be written once and students asked to prescribe IVT on a separate sheet (and asked not to write on the master copy!) |

*Example of template history for IVT prescribing (would need input/output chart in addition):*


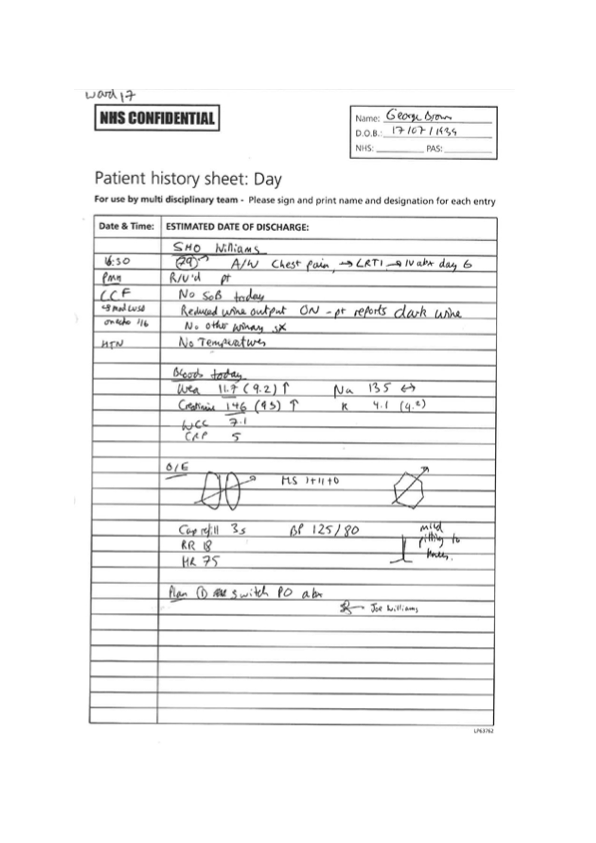


| **Painkiller prescribing** | |
| --- | --- |
| Task description: | Student asked by nurses to prescribe pain-killer for a patient |
| Materials required: | - Pre-filled drug chart with patient’s current medications:   - Paracetamol 1g QDS PRN (shown to be recently administered)   - Ramipril 5mg OD   - Bisoprolol 5mg OD - Student told limited history/nature of pain over the phone - mild, osteoarthritis flare up, ache in the right hip, no red flags. |
| Outcome: | - Students should first consider Ibuprofen as the most appropriate painkiller to address the nature of the pain, however should recognise the interactions with both ramipril and bisoprolol as being reasons why it may not be appropriate. Codeine 15mg PRN would be an appropriate next step. - Good students should also consider making paracetamol regular instead of PRN. - STAT doses shouldn’t be prescribed in this instance as the patient may need additional PRN relief. - No need to hand over in the morning |
| Comments: | Drug charts can be easily written up with the required information, and a master copy with photocopies can be made of the relevant pages to scale up to larger groups. It is helpful if it appears that some doses have already been given, particularly of the paracetamol. |

| **Agitated patient** | |
| --- | --- |
| Task description: | Student asked by nurses to prescribe sedative for a patient |
| Materials required: | - One continuation sheet with history of presenting complaint, some background past medical history/social history and examination:   - Patient is not acutely ill/is medically fit for discharge   - Patient is usually more confused at night.   - No falls/HI documented   - Nurse documents wants a sedative prescribed - Nurse insistent on the phone that sedative required |
| Outcome: | - Student should recognise and document that impression is confusion likely secondary to unfamiliar environment, Not UTI, not HI, not infection. - Suggest plan for conservative measures only - Only need to hand over if tests have been requested. - Ask student to reflect – how did the provided documented summary with patient baseline etc. help them? (importance of good documentation and regular summaries in notes) |
| Comments: | A good/thorough sheet with patient details should be provided, enabling the student to confidently come to the conclusion that the patient is confused secondary to an unfamiliar environment, rather than any other cause. A master copy of this could be made, leaving date/time blank (individual copies filled in to represent today’s date/time), and students should be asked to document their plan underneath this with what they would like to do (as if they were on call). |

*Example of template for agitated patient:*


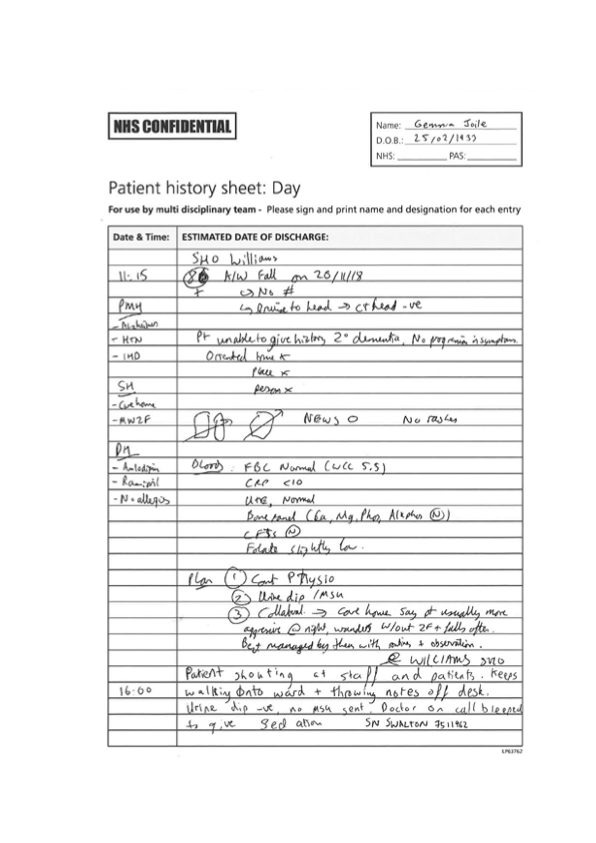


| **Xray review** | |
| --- | --- |
| Task description: | Student asked by nurses to review an X-ray by day team F1 |
| Materials required: | - Chest X-ray - Continuation sheet |
| Outcome: | - Student should interpret X-ray and document findings on a continuation sheet. - Plan suggested where relevant - Findings/jobs handed over to next day. |
| Comments: | A very simple exercise to make with multiple variations upon (different X-rays, ECGs, radiology reports etc) X-ray master copy with patient demographics and blank continuation sheet |

| **Kardex transcription** | |
| --- | --- |
| Task description: | - Student asked by nurses to re-write a drug chart |
| Materials required: | - Old drug chart, pre-filled. Suggestions: (not all need be implemented)   - Ibuprofen/bisoprolol - asthma interaction (patient on inhalers)   - PRN pain-killer used a lot, no regular pain relief (or on M/R morphine)   - Omeprazole/antibiotics interaction   - ACEI/NSAID interaction   - Been on 2 days IV antibiotics - New drug chart, blank - Nurse handover - patient well, on antibiotics, no recent temperatures. |
| Outcome: | - Students should:   - Recognise interactions and stop relevant medications     - Stop ibuprofen, replace with alternative painkiller     - Mark bisoprolol for review     - Increase BG painkiller/start regular painkiller     - Hold PPI, start ranitidine whilst on antibiotics     - Switch to PO antibiotics   - Maintain original starting dates for transcription medications rather than today’s dates.   - Write legal prescriptions - No need to hand-over |

South Tyneside District Hospital. ​*Authors: L. Baxter, A. Moxley, P. White* 28

| Comments: | This is slightly more time-consuming for students, and whilst some hospitals may have e-prescribing, making this more redundant, we feel this exercise is great for making students think about multiple areas!  A master copy drug chart can be made and photocopies of it printed to scale up. It may be worth initialling the nursing-boxes showing medications have been given to increase authenticity. |
| --- | --- |

**Other suggestions for on-call activities:**

- Warfarin prescribing (similar set-up to insulin)
- Anti-sickness (similar set up to pain-killer - consider passing on details of sickness secondary to bowel obstruction (metoclopramide contra-indicated) only if the student asks over the phone - this can be discussed in debrief)
- Follow up bloods/radiology (students bleeped by lab or given blood reports/radiology reports by teaching fellow with details of a patient from that day in the narrative. Task would be to action this on a continuation sheet)
- Canula arm (practice)
- ECG interpretation (similar set up to Xray report, you may want to include brief history to give nature of the pain)

Appendix E - Simulation resources

Below is an example scenario we used for our UGI bleed simulation.

Further simulation scenarios can be found by accessing the following eposter authored by Lucy Baxter on the ASME and AMEE websites from August 2019: Simulation in a District General Hospital; a six-month evaluation shows the juice is worth the squeeze.

| **Scenario Overview** | | |
| --- | --- | --- |
| **Title** GI bleed | | |
| **Original Design; Date** Lucy Baxter; 15/06/18 | | |
| **Reviewed/revised by:** Adam Moxley; 01/10/18 – adapted for final year medical students | | |
| **Estimated Running Time** 15 mins | | |
| **Target Learners** Final year medical students | | |
| **Case Summary** | Jonathan (Johnny) Walker is a 49-year-old man from South Shields. He is a known alcoholic and was admitted for symptoms of withdrawal. He is on the CIWA pathway. The FY1 doctor was called to review overnight as he had dropped his blood pressure. His past medical history includes alcohol excess and hypertension. He is unemployed and smokes 50/day. He had one episode of small volume coffee-ground vomit yesterday. He will turn out to have a GI bleed. | |
| **Learning Objectives Curriculum Mapping** | | |
| By the end of this session learners will be able to:   - ​Demonstrate initial assessment and management of the acutely unwell patient using a systematic ABCDE approach. - ​Recognise and appropriately manage shock - ​Prescribe appropriate fluid resuscitation. - ​Refer clearly and appropriately to senior colleague using SBAR format. - ​Recognise GI bleed. - ​**Additional**​: Know when and how to assess Blatchford score and how this can be used. | | Professionalism |
|  |  | Assessment in acute settings (admissions and deteriorating patient) |
|  |  | Investigation in acute settings (admissions and deteriorating patient) and Core investigations |
|  |  | Treatment and management in acute settings (admissions and deteriorating patient) |
|  |  | Prescribing |
|  |  | Communication in acute settings (admissions and deteriorating patient) |
|  |  | Situational awareness, personal organisation, and teamwork skills in acute settings (admissions and deteriorating patient) |
| **Scenario Set Up / Initial Appearance / Equipment** | | |
| **Clinical Setting** Ward 3 (gastroenterology) | | |
| **Place** Simulation room | | |
| **Simulator** Sim Man 3G | | |
| **Equipment /Props** Resuscitation trolley, IV fluid, syringes, phone, cannulation trolley | | |
| **Documents** | **Completed documents** | A+E clerking, EAU clerking, ward round entry, drug chart, CIWA chart, EWS chart |
|  | **Blank documents** | N/A |
|  | **Investigations**  Admission bloods, admission CXR, admission ECG | |
|  | **Investigations available on request** | ABG/ VBG |
| **Initial Mannequin Set Up** | **Position**  Semi reclined | |
|  | **Moulage**  n/a | |
|  | **Attire**  Hospital gown | |
|  | **Catheters tubes & lines** | 1 cannula |
|  | **Monitoring**  HR 125, BP 90/75, RR 24, Sats 93% on air | |
|  | **Infusions**  N/A | |

South Tyneside District Hospital. ​*Authors: L. Baxter, A. Moxley, P. White* 31

| **Information for Participants Voice of Patient:** | |
| --- | --- |
| The nurse has bleeped you about a patient on ward 3 who they are unsure about but think is very sick. | You are confused and tired. You just keep saying “what’s going on?” and “what you doing?” You can say you’re name and age (Jonathan (Johnny) Walker, 49) and you do not have pain but to most other questions you either do not respond as you have dropped off (you will wake to loud voice/shaking shoulder) or say “I dunno.” |
| **Confederate: Additional Faculty:** | |
| You are a student nurse. You bleeped the doctor as you are very worried about this patient. Their BP has dropped and they seem drowsy. You’re very pleased when the doctor arrives, you really are worried. You are eager to help- you will do what they say without hesitation. | **Medical Registrar:** ​You want to know their clinical findings, BP and HR, risk factors and ask for Blatchford score – prompt the student to calculate it when you’re on your way if they have not calculated it already. You agree to review the patient after the SBAR handover. |

| **Stage/Event** | **State** | **Expected Behaviours** | **Transitions and Prompts** | **Potential Teaching Points** |
| --- | --- | --- | --- | --- |
| **Baseline**  Shocked patient | A - patent  B- RR24,Sats 94% on air, chest clear  C- HR125,BP 90/75, HS normal | ABCDE assessment. Recognises shock. Gives fluid challenge (Hartmans or saline) Considers differential diagnoses for shock in systematic way. | **Trigger**  Recognises shock and considers differential diagnoses  *Physiology:* n​ /a  *Patient:*   1. ​“What’s going on?” 2. ​“I feel dizzy”   *Faculty:*  1)​ ​“Their BP is pretty low” | Assessment of shocked patient. Differential diagnoses of shocked patient. |
|  | D- confused, alert to voice, BM 4.1  E – Temp 36.7 |  | 2)​ ​“What do you think could be going on?” 3)​ ​“What other reasons are there they could be shocked?” |  |
| **State 2**  Shocked patient | A - patent  B- RR30,Sats 93% on air, chest clear  C- HR125,BP 90/75, HS normal  D- confused, alert to voice, BM 3.9  E – Temp 37.3 | Recognises melaena and diagnoses UGI bleed. Cross matches blood. Puts a second wide bore cannula in. | **Trigger**  Recognises melaena and cross matches blood.  *Physiology:* n​ /a  *Patient:* n​ /a  *Faculty:*   1. 1)​  ​“What’s that smell?” 2. 2)​  ​“I think this is   melaena” (lifts up sheets) | Management of GI bleed. Risk factors for GI bleed. |
| **State 3**  Shocked patient | A - patent  B- RR30,Sats 93% on air, chest clear  C- HR125,BP 90/75, HS normal  D- confused, alert to voice, BM 3.9 | Discusses with medical registrar using SBAR handover.  Additional: Calculates Blatchford score. | **Trigger**  Discusses with medical registrar.  *Physiology: Patient:* n​ /a  *Faculty:*  n/a | Blatchford score. Assessment of blood loss/severity of haemorrhage. |
|  | E – Temp 37.3 |  | 1)​ ​“Do you think you need to ring the registrar?” |  |
| **State 4**  Shocked patient | A - patent  B- RR30,Sats 93% on air, chest clear  C- HR125,BP 90/75, HS normal  D- confused, alert to voice, BM 3.9  E – Temp 37.3 |  | **Trigger for scenario to end** Medical registrar arrives to review patient. | SBAR handover. Capacity re: endoscopy |
|  |  |  | *Physiology:*  n/a  *Patient:*  *Faculty*:​ n/a  n/a |  |
